# Novel genetic variants of inborn errors of immunity

**DOI:** 10.1371/journal.pone.0245888

**Published:** 2021-01-22

**Authors:** Farida Almarzooqi, Abdul-Kader Souid, Ranjit Vijayan, Suleiman Al-Hammadi

**Affiliations:** 1 Department of Pediatrics, College of Medicine and Health Sciences, UAE University, Al Ain, United Arab Emirates; 2 Department of Biology, College of Science, UAE University, Al Ain, United Arab Emirates; Unicamillus, Saint Camillus International University of Health Sciences, ITALY

## Abstract

**Objectives:**

Inborn errors of immunity (IEI) are prevalent in tribal cultures due to frequent consanguineous marriages. Many of these disorders are autosomal recessive, resulting from founder mutations; hence they are amenable to prevention. The primary objective of this study was to evaluate the pathogenicity of novel variants of IEI found among Emiratis.

**Methods:**

This retrospective data collection study reports novel variants of IEI detected by diagnostic exome sequencing. Pathogenicity prediction was based on scoring tools, amino acid alignment, and Jensen–Shannon divergence values.

**Results:**

Twenty-one novel variants were identified; nine were frameshift, three nonsense, four intronic (one pathogenic), and five missense (two pathogenic). Fifteen variants were likely pathogenic, of which 13 were autosomal recessive and two uncertain inheritance. Their clinical spectra included combined immunodeficiency, antibody deficiency, immune dysregulation, defects in intrinsic/innate immunity, and bone marrow failure.

**Conclusion:**

The described novel pathogenic variants are core to a planned national screening program that aims toward IEI prevention. Future studies, however, are needed to confirm their natural history in individual patients and estimate their prevalence in the community.

## Introduction

There are over 400 genes implicated in inborn errors of immunity (IEI) [1]. Identifying their variants is critical for patient care, genetic prevention, and translational research [2]. For this purpose, diagnostic exome sequencing has proven helpful in preventing primary immunodeficiency (PID) in tribal populations where founder mutations and autosomal recessive (AR) disorders are exceptionally common [3].

A national premarital screening program was implemented in the United Arab Emirates (UAE) in 2005 (Personal Status Act No 28, Article 27). This endeavor has eliminated sickle cell disease and beta-thalassemia major from the community. This study examines indigenous (novel) pathogenic variants of IEI, and suggests including them in the existing premarital screening program.

## Methods

This retrospective data collection (Chart Review) study was approved through the ‘Tawam Human Research Ethics Committee’ (T-HREC); reference number: MF2058-2020-722 (AA/AJ/722; May 13, 2020), and informed consent to participate was waived. The study involved one center (Tawam Hospital, Al Ain City, Abu Dhabi, UAE). A review of 54 variants of IEI (detected by diagnostic exome sequencing between March 2016 and November 2019) was conducted between May 13, 2020 and July 10, 2020. Of these, 21 variants were novel and included in this report. Thirteen variants involved patients and eight were found on a screening test.

Variant information in public databases was consolidated using Ensembl Variant Effect Predictor (https://www.ensembl.org/vep). Gathered data included effect of variation, codon change, amino acid change, and variations from dbSNP, functional consequence, and pathogenicity score from algorithms, such as SIFT, PolyPhen, Condel, CADD, FATHMM, LRT, MetaLR, MetaSVM, MutationAssessor, MutationTaster, PROVEAN, REVEL, and VEST3 in dbNSFP (One-Stop Database of Functional Predictions and Annotations for Human Non-synonymous and Splice Site). The Mutation Significance Cutoff (MSC) Server was used to obtain gene-wise CADD MSC [4].

Multiple sequence alignment was used to evaluate conservation of amino acids at studied sites and compute the Jensen-Shannon divergence (JSD) values. Amino acid sequences of proteins from *Homo sapiens* (human), *Pan troglodytes* (chimpanzee), *Mus musculus* (house mouse), *Rattus norvegicus* (Norway rat), *Canis lupus familiaris* (dog), *Equus caballus* (horse), *Bos taurus* (bovine), *Xenopus tropicalis* (frog), *Gallus gallus* (chicken), and *Danio rerio* (zebrafish) were downloaded from NCBI RefSeq (S1 Table). Sequences were imported into Geneious 9.1.8 (https://www.geneious.com) using NCBI Accessions and multiple sequence alignment was performed using MUSCLE. Alignments were exported in FASTA format and used to obtain JSD values from “https://compbio.cs.princeton.edu/conservation/score.html” using the default options [5].

Homology model of the ATM (Ataxia telangiectasia mutated) protein with the variation (*ATM*:p.Val2823Gly) was generated using the SWISS-MODEL server (https://swissmodel.expasy.org/). The Protein Data Bank (PDB) structure 6K9L (4.27 Angstrom resolution cryo-EM structure of human dimeric ATM kinase) was used as the template. Protein structures were visualized using Visual Molecular Dynamics 1.9.3 [6].

### Statistics

Probability of having an autosomal recessive disorder(s) in the offspring was calculated on Excel, using binomial probability distribution (probability of having a recessive phenotype of 0.25 with variable number of genes).

## Results

Table 1 lists the 21 novel variants of genes (19 single genes) related to IEI, sorted according to the International Union of Immunological Societies Phenotypical Classification [1]. Thirteen variants were found in symptomatic patients, while eight variants were found on a screening test in asymptomatic subjects. Fifteen variants are autosomal recessive (AR, 71%), three undetermined inheritance, one autosomal dominant (AD), and two X-linked. Nine variants are frameshift, three nonsense, four intronic (one pathogenic), and five missense (two pathogenic). Table 2 shows sequence alignment of the proteins at positions of the missense variants from ten species.

**Table 1:** Novel variants of IEI.

*Variants*	*Phenotypes*	*SIFT*	*PolyPhen*	*Condel*	*CADD (MSC)*	*FATHMM*	*LRT*	*MetaLR*	*MetaSVM*	*Mut Assessor*	*Mut Taster*	*PROVEAN*	*REVEL*	*VEST3*	*Predicted Pathogenicity*
**Severe combined immunodeficiency (CD3 T-cell count<300/μl)**															
NM_001033855.3(*DCLRE1C*):c.545G>A, p.Cys182Tyr DNA cross-link repair protein 1C; MIM#605988; missense; [tGt]>[tAt]	Omenn syndrome (MIM# 603554) and severe combined immunodeficiency, Athabascan type (MIM#602450); AR	0.00 D	0.993 D	0.895 D	25.3 (11.57)	0.797	0.843	0.875	0.889	0.694	0.588	0.899	0.868	0.886	Pathogenic
**Combined immunodeficiency with low CD8**															
NM_000544.3(*TAP2*):c.753dupA, p.Arg252ThfsTer11 Transporter, ATP-binding cassette, major histocompatibility complex; MIM#170261; frameshift; [–]>[A]	Bare lymphocyte syndrome, type I, due to TAP2 deficiency; MIM#604571; AR	-	-	-	-	-	-	-	-	-	-	-	-	-	Pathogenic
**Combined immunodeficiency with DNA repair defects**															
NM_000051.3(*ATM*):c.8468T>G, p.Val2823Gly Ataxia-telangiectasia mutated gene; MIM#607585; missense; [gTt]>[gGt]	Ataxia-telangiectasia, AT; MIM#208900; AR	0.00 D	0.741 D	0.716 D	28.5 (6.38)	0.757	0.513	0.834	0.855	0.612	0.810	0.817	0.944	0.789	Pathogenic
**Combined immunodeficiency with syndromic features**															
NM_138576.3(*BCL11B*):c.767G>A, pSer256Asn BAF chromatin remodeling complex subunit BCL11B; MIM#606558; missense; [aGc]>[aAc]	Immunodeficiency 49 (MIM#617237) and intellectual developmental disorder with dysmorphic facies, speech delay, and T-cell abnormalities (MIM#618092); AD	0.34 T	0.084 B	0.029 N	23.9 (10.67)	0.453	0.255	0.201	0.088	0.045	0.411	0.212	0.080	0.068	Benign
NM_203447.3(*DOCK8*):c.3339dup, p.Met1114TyrfsTer4 Dedicator of cytokinesis 8; MIM#611432; frameshift; [–]>[T]	Hyper-IgE recurrent infection syndrome 2; MIM#243700; AR	-	-	-	-	-	-	-	-	-	-	-	-	-	Pathogenic
NM_032790.3(*ORAI1*):c.454C>A, p.His165ProfsTer2 ORAI calcium release-activated calcium modulator 1; MIM# 610277; frameshift; [ccc]>[cCcc]	Combined immunodeficiency due to ORAI1 deficiency; MIM#612782; AR	-	-	-	-	-	-	-	-	-	-		-	-	Pathogenic
NM_080424.2(*SP110*):c.691C>T, p.Gln231Ter Nuclear body protein SP110; MIM#604457; nonsense; [Caa]>[Taa]	Hepatic venoocclusive disease with immunodeficiency; MIM#235550; AR	-	-	-	7.97 (12.46)	-	-	-	-	-	0.810	-	-	-	Pathogenic
**Antibody deficiency**															
NM_001287344.1(*BTK*):c.80G>A, p.Gly27Asp Bruton agammaglobulinemia tyrosine kinase; MIM#300300; missense; [gGt]>[gAt]	X-linked agammaglobulinemia; MIM#300755	-	0 B	-	12.49 (10.1)	-	-	-	-	-	0.320	-	-	-	Benign
**Defects in phagocytes**															
NM_001145873.1(*CD8A*):c.49+2T>G CD8 Antigen, alpha polypeptide; MIM#186910; splice-donor	CD8 deficiency, familial; MIM#608957; AR	-	-	-	28.1 (8.93)	-	-	-	-	-	-	-	-	-	Pathogenic
NM_156039.3(*CSF3R*):c.1015delG, p.Asp339ThrfsTer94 Colony-stimulating factor 3 receptor, granulocyte; MIM#138971; frameshift; [Gac]>[ac]	Neutropenia, severe congenital, 7, autosomal recessive; MIM#617014; AR	-	-	-	-	-	-	-	-	-	-	-	-	-	Pathogenic
**Immune dysregulation**															
NM_006726.4(*LRBA*):c.974C>T; p.Ala325Val Lipopolysaccharide-responsive, beige-like anchor; MIM#606453; missense; [gCt]>[gTt]	Immunodeficiency, common variable, 8, with autoimmunity; MIM#606453; AR	0.62 T	0.145 B	0.017 N	26.7 (33)	0.62918	0.62929	0.52904	0.48446	0.04202	0.58761	0.27052	0.40608	0.84713	Benign
NM_006726.4(*LRBA*):c.8007G>A; p.Trp2669Ter Lipopolysaccharide-responsive, beige-like anchor; MIM#606453; nonsense; [tgG]>[tgA]		-	-	-	32.0 (33)	-	0.843	-	-	-	0.810	-	-	-	Pathogenic
NM_006726.4(*LRBA*):c.534del, p.Asp179IlefsTer16 Lipopolysaccharide-responsive, beige-like anchor; MIM#606453; frameshift; [ggA]>[gg]		-	-	-	-	-	-	-	-	-	-	-	-	-	Pathogenic
**Defects in intrinsic and innate immunity**															
NM_001127593.1(*FCGR3A*):c.423dupT, p.Leu142PhefsTer13 Fc fragment of IgG, low affinity IIIa, receptor for; MIM#146740; frameshift; [–]>[T]	Immunodeficiency 20; MIM#615707; AR	-	-	-	-	-	-	-	-	-	-	-	-	-	Pathogenic
NM_005534.3(*IFNGR2*):c.123C>G, p.Tyr41Ter Interferon-gamma receptor 2; MIM#147569; nonsense; [taC]>[taG]	Immunodeficiency 28; MIM#614889; AR	-	-	-	35.0 (4.43)	-	0.315	-	-	-	0.810	-	-	-	Pathogenic
**Hemophagocytic lymphohistocytosis (HLH) and Epstein-Barr virus (EBV) susceptibility**															
NM_032121.5(*MAGT1*):c.198+383G>C Magnesium transporter 1; MIM#300715; splice-donor	Immunodeficiency, X-linked recessive, with magnesium defect, EBV virus infection and neoplasia; MIM#300853	-	-	-	-	-	-	-	-	-	-	-	-	-	Benign
**Bone marrow failure**															
NM_007300.3(*BRIP1*):c.4358-2A>T BRCA1-interacting protein 1; MIM#605882; splice-acceptor	Fanconi anemia, complementation group J; MIM#609054; unknown inheritance	-	-	-	-	-	-	-	-	-	-	-	-	-	Uncertain
NM_033084.4(*FANCD2*):c.1275_1278+5delCTTAGTAAGinsTTTAT, p.Tyr425_Leu426delins FANCD2 gene; MIM#613984; splice donor	Fanconi anemia, complementation group D2; MIM#227646; AR	-	-	-	-	-	-	-	-	-	-	-	-	-	Uncertain
NM_001113378.1(*FANCI*):c.1289_1290delTT, p.Phe430fsTer FANCI gene; MIM#611360; frameshift; [tTT]>[t]	Fanconi anemia, complementation group I; MIM#609053; AR	-	-	-	-	-	-	-	-	-	-	-	-	-	Pathogenic
**Others**															
NM_002641.3(*PIGA*):c.1077_1078delinC, p.Leu359PhefsTer19 Phosphatidylinositol glycan anchor biosynthesis class a protein; MIM#311770; frameshift; [ttGGaa]>[ttaa]	Paroxysmal nocturnal hemoglobinuria, somatic; MIM#300818; unknown inheritance	-	-	-	-	-	-	-	-	-	-	-	-	-	Pathogenic
NM_018676.3(*THSD1*):c.1322_1329del, p.Arg441GlnfsTer66 Thrombospondin type 1 domain-containing protein 1; MIM#616821; frameshift; [cGGCCAGCC]>[c]	Aneurysm, intracranial berry, 12; MIM#618734; unknown inheritance	-	-	-	-	-	-	-	-	-	-	-	-	-	Pathogenic

SIFT (Range: 0–1; “Deleterious (D)” if score is less than 0.05; “Tolerated (T)” if score is greater than or equal to 0.05), PolyPhen-2 (Range: 0–1; “Probably Damaging (D)” if score is greater than 0.908; “Benign (B)” if score is less than or equal to 0.446), Mutation Assessor, Mutation Taster (integrates scores from Ensembl, UniProt, ClinVar, ExAC, 1000 Genomes Project, phyloP, and phastCons), LRT (likelihood ratio test, based on a probabilistic estimation of the phylogenetic relationship, with residue changes treated equally rather than weighting radical or conservative amino acid changes differently), PROVEAN (protein variation effect analyzer) and FATHMM (functional analysis through hidden Markov models) are pathogenicity prediction based on alignment of protein sequences and/or structures. Condel (consensus deleterious) is a consensus prediction based on SIFT and PolyPhen-2 scores. REVEL (rare exome variant ensemble learner), CADD (combined annotation‐dependent depletion; scores range from 1 to 99, e.g., a score of 30 signifies a 0.1% top variant), MSC (Mutation Significance Cutoff at the lower boundary of the 95% confidence interval for the CADD score of the gene; the phenotypic impact is possibly damaging above this cutoff), MetaLR (meta-analytic logistic regression) and MetaSVM (meta-analytic support vector machine) are ensemble-based prediction based on multiple deleteriousness, conservation and ensemble scoring methods derived from surrounding sequences, gene-model interpretations, evolutionary constraints, epigenetic extents, pathogenicity predictions and allele frequencies. B, benign; D, deleterious; T, tolerated; AR autosomal recessive; AD, autosomal dominant.

**Table 2:** Sequence alignment of protein sequences at positions of the missense variants with the Jensen-Shannon divergence (JSD) values.

	*ATM*:p.Val2823Gly	*BCL11B*:pSer256Asn	*BTK*:p.Gly27Asp	*DCLRE1C*:p.Cys182Tyr	*LRBA*:p.Ala325Val
Human	Val	Ser	Gly	Cys	Ala
Chimpanzee	Val	Ser		Cys	Ala
Mouse	Ile	Ser		Cys	Ala
Rat	Ile	Ser		Cys	Ala
Dog	Ile	Ser		Cys	Ala
Horse	Ile	Ser	Ser	Cys	Ala
Bovine	Ile	Ser		Cys	Ala
Frog	Ile	Ser		Cys	Ala
Chicken	Val	Asn	Glu	Cys	Ala
Zebrafish	Val	Ser		Cys	Ala
**JSD value**	0.7041	0.7603		0.9189	0.7990

### Two variants involve combined immunodeficiency

*DCLRE1C*:p.Cys182Tyr is predicted to be pathogenic based on the *in silico* prediction scores (Table 1) and phylogenetic analysis (Cys182 is conserved; JSD: 0.919, Table 2). *TAP2*:p.Arg252ThrfsTer11, which results in the truncation in the ABC transporter transmembrane domain making it non-functional, is likely to cause combined immunodeficiency with low CD8 [7]. A homozygous truncation at position 253 of TAP2, leading to a non-functional TAP2, has also been demonstrated to be associated with human lymphocyte antigen (HLA) class I deficiency [8].

### One variant involves ataxia-telangiectasia (AT)

**Fig 1:**
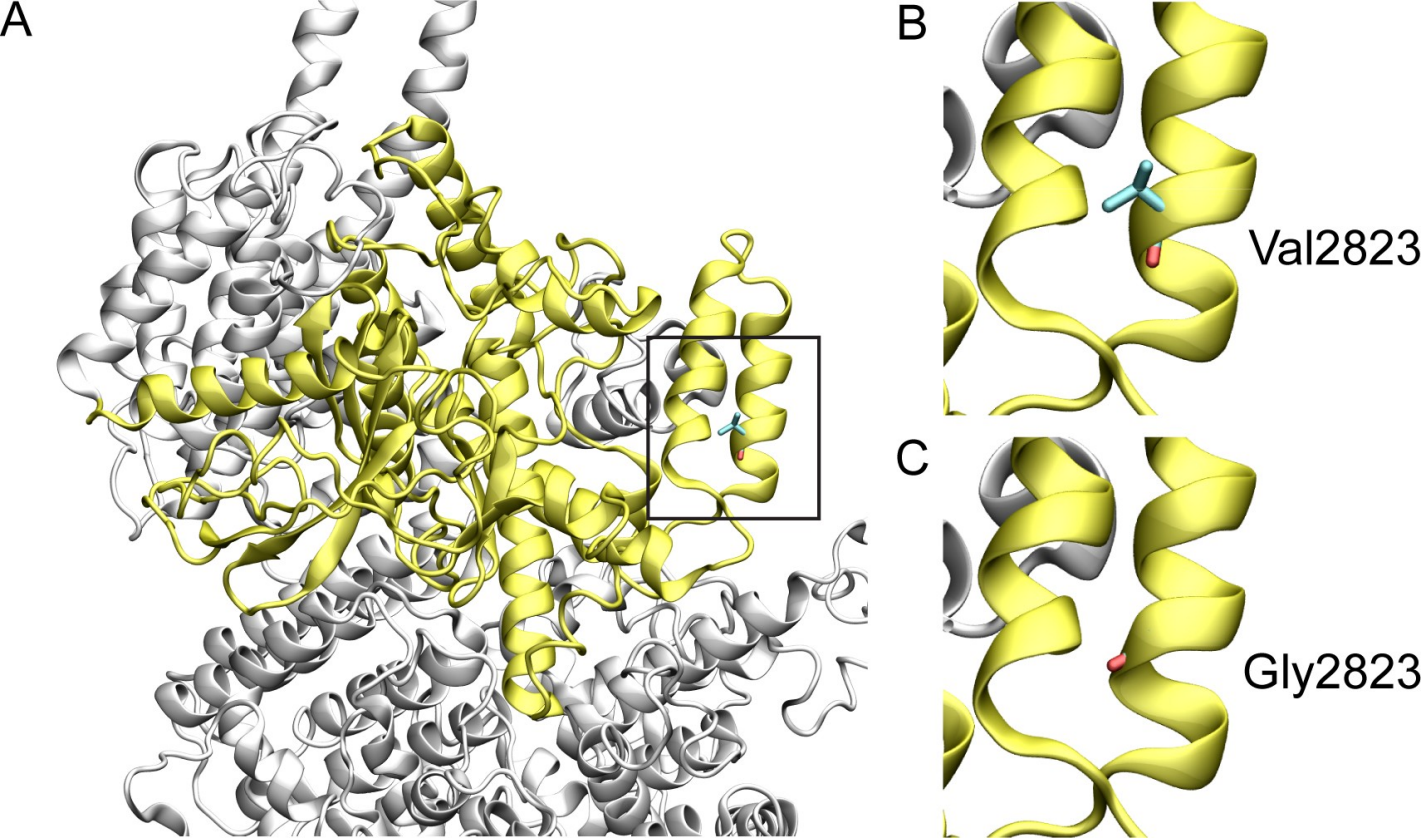
Structural model of wild-type and variant ATM protein. The ATM protein structure is shown in white cartoon representation and the kinase domain is shown in yellow. Amino acids are shown in stick representation. The black boxed region is enlarged in the subsequent two images. (**A**) Structure of the conserved kinase domain of ATM, (**B**) wild type Val2823, and (**C**) variant Gly2823.

*ATM*:p.Val2823Gly has conflicting predictions of pathogenicity. Val2823 is conserved, with a permissible change to isoleucine (JSD: 0.7041, Table 2). Val2823 (Fig 1) is located in the kinase domain of the alpha helical C terminus. Gly2823 is predicted to disturb the stability of the helical conformation, thus, affecting the structure and function of the protein [9,10]. This assessment is consistent with the SIFT score of 0.00 (damaging) and the CADD score of 28.9 (Table 1).

### Three variants involve combined immunodeficiency with syndromic features

*BCL11B*:pSer256Asn has benign predictions, except for CADD: 23.9. Ser256 is replaced by asparagine in chicken (Table 2), supporting the benign predictions. *DOCK8* variant p.Met1114TyrfsTer4 is likely pathogenic since the truncation resulting from the frameshift leads to the deletion of the DOCKER domain that is essential for the guanine nucleotide exchange factor activity of DOCK8. *ORAI1*:p.His165ProfsTer5, a truncation that results in the deletion of two transmembrane helices of the membrane protein, and *SP110*:p.Gln231Ter, a truncation in the transcription factor, are also predicted to be pathogenic. Of note, in this category, two microdeletions (2.55 Mb and 3.152 Mb) within 22q11.21 were found; they cause *TBX1* haploinsufficiency and hence are disease-causing (DiGeorge syndrome) [11].

### One variant involves antibody deficiency

Variant *BTK*:p.Gly27Asp is benign (Table 1). Gly27 is replaced by glutamate in chicken (Table 2).

### Two variants involve phagocyte defects

*CD8A*:c.49+2T>G (CADD: 28.1) and *CSF3R*:p.Asp339ThrfsTer94, in the third fibronectin type III domain in this protein is likely to cause it to misfold, and hence likely pathogenic. *CSF3R* encodes the G-CSF (granulocyte colony-stimulating factor) receptor, essential for JAK/STAT-mediated granulopoiesis [12].

### Variants of LRBA involve immune dysregulation

*LRBA*:p.Ala325Val has benign scores, except for CADD: 26.7 and VEST3: 0.847 (Table 1). Ala325 is conserved (JSD: 0.799, Table 2). The *LRBA* variants p.Trp2669Ter (CADD: 32), affects the third WD repeat (short motifs often terminating at Trp-Asp) and likely impacts the folding of this region, and p.Asp179IlefsTer16, which results in the deletion of the BEACH domain and the WD repeats, are both likely to be pathogenic.

### Two variants involve intrinsic/innate immunity

*FCGR3A*:p.Leu142PhefsTer13, in the second immunoglobulin-like domain, and *IFNGR2*:p.Tyr41Ter (CADD: 35), in the first fibronectin type III domain, are both likely to affect the structure and function of these protein and hence likely pathogenic.

### One variant involve hemophagocytic lymphohistocytosis and Epstein-Barr virus susceptibility

*MAGT1*:c.198+383G>C is benign.

### Three variants involve bone marrow failure syndromes

*BRIP1*:c.4358-2A>T and *FANCD2*:c.1275_1278+5delCTTAGTAAGinsTTTAT are intronic with uncertain clinical significance. *FANCI*:p.Phe430fsTer results in a truncation that deletes the DNA binding region of a protein associated DNA repair; it is therefore likely pathogenic.

*PIGA*:p.Leu359PhefsTer19, associated with the glycosyl transferases group 1 family, is likely pathogenic causing paroxysmal nocturnal hemoglobinuria. *THSD1*:p.Arg441GlnfsTer66, resulting from an eight-base deletion, is also likely pathogenic.

## Discussion

This study describes 21 novel variants of IEI. Their types include frameshift, nonsense, intronic, and missense. Our bioinformatics analysis shows the majority (15 of 21, or 71%) are pathogenic or likely pathogenic; of these, 13 are autosomal recessive. Thus, these mutations are amenable for genetic prevention via screening and counseling. Future studies, however, are needed to confirm their prevalence and natural history in individual patients.

The variants reported here are novel. Their prediction of pathogenicity is assessed by *in silico* computer tools described above. Thus, functional validation of these variants remains fundamental to attribute pathogenicity with certainty [13,14]. As previously shown for cancer predisposition, improved genomic analysis lowers the prevalence of variant of uncertain significance (VUS) [15]. The authors concluded that variant “interpretation by multiple experts in the context of personal and family histories maximizes actionable results and minimizes reports of VUS” [15]. Similar efforts are needed for novel variants of IEI.

Pathogenic variants of autosomal recessive disorders carry ‘fetal risk’ for homozygosity and compound heterozygosity, especially in tribal cultures with frequent consanguineous marriages. Fetal risk is, of course, higher in the presence of multiple pathogenic variants of a gene, especially within a tribe, such as the three variants of *LRBA* (Table 1). Another example is the six known pathogenic variants of ataxia telangiectasia (AT) in our community; one of these is novel and reported here. This entity is characterized by genomic instability due to defects in DNA repair [16]. Homozygous and double heterozygous affected individuals have immune derangements (e.g., thymic involution) and central nervous system degeneration. Heterozygous individuals may show mild clinical features, such as increased risk for cancer and hypersensitivity to radiation [17,18]. Thus, the AT variants carry an exceptional fetal risk for homozygosity and compound heterozygosity, especially in our tribal cultures that mainly practice consanguineous marriages. Such variants need to be built into a national screening program aiming at disease prevention [19].

Generation of homology models is often hampered by the unavailability of wild-type human or homologous protein structures. Hence, in this instance, the only variant that could be modelled was the ATM:p.Val2823Gly based on the 4.27 Å cryo-electron microscopy structure of ATM (PDB ID: 6K9L). Val283 is part of the helix designated as kα4c in the phosphatidylinositol 3-/4-kinase catalytic domain of the ATM serine-protein kinase [20]. This valine, or the physiochemically similar isoleucine, is conserved in all species considered here. While the precise role of the helix is not known, a change to a glycine, a notably achiral amino acid, at this position could potentially disrupt the stability of this helix.

Twenty-one IEI novel variants are categorized based on the IUIS Phenotypical Classification [1]. This allows simpler dissection of IEI molecular diagnosis in association with clinical and immunological profiles. It serves as a framework to determine IEI types and prevalence in the UAE. It will also enhance our immunological understanding of the variant to aid patient care with targeted therapy and prevention with family screening and counselling.

The novel IEI variants reported here include one variant of severe combined immunodeficiency (SCID); two variants of combined immunodeficiency (CID) with low CID and DNA repair defect; four variants of CID with syndromic features; one variant of antibody deficiency; two variants of defects in phagocytes; three variants of one gene related to immunodysregulation; two variants of defects in intrinsic and innate immunity; one variant of hemophagocytic lymphohistocytosis (HLH) and Epstein-Barr virus (EBV) susceptibility; three variants related to bone marrow failure; and two variants under ‘others’. PIGA and THSD1 variants under ‘Others’ are not reported in IUIS. However, the PIGA variant causes paroxysmal nocturnal hemoglobinuria (PNH) and is associated with cellular immunodeficiency. It is hypothesized that PNH impacts lymphocyte count through decrease in lymphopoiesis and complement-mediated damage [21]. A case series in UAE shows the THSD1 variant causes intracranial berry aneurysm 12, nonimmune hydrops fetalis, congenital cardiac defects, hemangiomas and hypogammaglobulinemia [22].

One of the major limitations of diagnostic sequencing and genetic testing is that the effect of a variation on function cannot be inferred from sequence alone. Considering the volume of next generation data produced, a large proportion of variants currently remain classified as variants of uncertain significance (VUS) in bioinformatics databases. While phenotype data could address this problem partly, systematic *in vitro* functional and mutational assays are necessary to pinpoint the precise role of a variant on the structure and function of a protein and its pathways [14]. Often, a single assay may not be sufficient for this purpose elevating the difficulty in establishing the pathogenicity of VUS. Nonetheless, in this study, based on computational predictors, we have attempted to predict the pathogenicity of novel variants identified in our population. However, as indicated, functional assays would be necessary to ascertain the precise pathogenicity of these variants.

IEI are heterogeneous disorders and are often underdiagnosed. Knowing the causative genetic variant offers a better prediction of its course and opens up opportunities for participating in clinical trials related to the defect. Genetic screening, on the other hand, offers early identification of people at risk; thereby, allowing more prompt interventions [19]. Thus, genetic testing is required for all individuals with immunodeficiency. Our results will aid in setting the framework of a national screening program aiming at genetic prevention of IEI.

## Supporting information

S1 Table NCBI RefSeq accession of sequences used for protein sequence alignment. (PDF)
