# Supplementary material for: Novel genetic variants of inborn errors of immunity
Source: PLoS One. 2021 Jan 22;16(1):e0245888. doi: 10.1371/journal.pone.0245888 (PMC7822508; doi:10.1371/journal.pone.0245888)
Supplement: S1 Table — (PDF) [file pone.0245888.s001.pdf]

Novel Genetic Variants of Inborn Errors of Immunity

Farida Almarzooqi [1* ¶ &#a], Abdul-Kader Souid[1¶&#a], Ranjit Vijayan[2&#b], Suleiman Al Hammadi[1¶#a]

[1]Department of Pediatrics, College of Medicine and Health Sciences, UAE University

[2]Department of Biology, College of Science, UAE University

[#a] Current address: Department of Pediatrics, College of Medicine and Health Sciences, UAE University, Abu Dhabi, United Arab Emirates

[#b] Current address: Department of Biology, College of Science, UAE University, Abu Dhabi, United Arab Emirates

[*]Corresponding author: Farida Almarzooqi - E-mail: famarzooqi@uaeu.ac.ae

Table S1: NCBI RefSeq Accession of sequences used for protein sequence alignment

**Gene**	**Protein**	***Homo sapiens* (Human)**	***Pan troglodytes* (Chimpanzee)**	***Mus musculus* (Mouse)**	***Rattus norvegicus* (Rat)**	***Canis lupus familiaris* (Dog)**	***Equus caballus* (Horse)**	***Bos taurus* (Bovine)**	***Xenopus tropicalis* (Frog)**	***Gallus gallus* (Chicken)**	***Dano rerio* (Zebrafish)**
ATM	ATM	NP_000042.3	XP_024203167.1	XP_011240687.1	NP_001100291.1	NP_001124300.1	XP_014596526.2	XP_010810627.1	XP_002934974.3	XP_015133057.1	XP_002664603.3
BCL11B	BCL11B	NP_612808.1	XP_001151763.2	XP_011242454.1	NP_001264216.1	XP_005623848.1	XP_023484130.1	XP_002696813.3	XP_004917173.1	XP_003641458.1	XP_002667906.3
BTK	BTK	NP_001274273.1	XP_024209015.1	NP_038510.2	NP_001007799.1	XP_005641623.1	XP_023490284.1	NP_001029761.1	NP_001123732.1	XP_015133649.1	XP_021336920.1
DCLRE1C	Artemis	NP_001029027.1	XP_024201986.1	NP_666226.2	XP_017455949.1	XP_013977953.1	XP_001498525.3	XP_010809416.1	XP_002935298.1	NP_001026765.1	XP_021330740.1
LRBA	LRBA	XP_005263430.1	XP_016807843.1	XP_006502411.1	NP_001102025.1	XP_013974839.1	XP_023483491.1	XP_024833308.1	XP_002933545.3	XP_015132156.1	XP_009289517.1
